# A Novel Prognostic Nomogram for Predicting Survival of Hormone Receptor-Positive and HER2 Negative Advanced Breast Cancer Among the Han-Population

**DOI:** 10.3389/fonc.2022.918759

**Published:** 2022-07-01

**Authors:** Yimin Zhu, Jiayu Wang, Binghe Xu

**Affiliations:** Department of Medical Oncology, National Cancer Center/National Clinical Research Center for Cancer/Cancer Hospital, Chinese Academy of Medical Sciences and Peking Union Medical College, Beijing, China

**Keywords:** advanced breast cancer, hormone receptor, HER2 status, nomogram, survival prediction

## Abstract

**Purpose:**

To develop a nomogram model to predict overall survival in HR+/HER2- subtype advanced breast cancer.

**Methods:**

A total of 3,577 ABC (advanced breast cancer) patients from 21 hospitals in China were involved in this study from January 2012 to December 2014. From all ABC patients, 1,671 HR+/HER2- ABC patients were extracted and enrolled in our study. A nomogram was built based on univariable and multivariable Cox regression analyses, identifying independent predictors. The discriminatory and predictive capacities of the nomogram were assessed using the ROC (receiver operating characteristic) curve and calibration plots.

**Results:**

Univariable and multivariable analysis found that ER (estrogen receptor) status, MFIs (metastatic-free intervals), first-line therapy options, the number of metastatic sites, and whether local therapy for metastatic sites was chosen, were significantly related to overall survival (all *P* < 0.05). These variables were incorporated into a nomogram to predict the 2- year, 3-year, and 5-year OS (overall survival) of ABC patients. The AUC (the area under the curve) of the nomogram was 0.748 (95% CI (confidence interval):0.693-0.804) for 5-year OS in the training cohort and 0.732 (95% CI: 0.676-0.789) for the validation cohort. The calibration curves revealed good consistency between actual survival and nomogram prediction in the training and validation cohorts. Additionally, the nomogram showed an excellent ability to stratify patients into different risk cohorts.

**Conclusion:**

We established a nomogram that provided a more straightforward predictive model for the outcome of HR+/HER2- ABC subtype patients and, to some extent, assisted physicians in making the personalized therapeutic option.

## Introduction

BC (breast cancer) is the most frequent cancer, with first incidence and second mortality in all malignant tumors among Chinese women and it accounts for about 15% of all new cancer cases every year in Chinese women (1, 2). Reportedly, approximately 30% of women diagnosed with EBC (early breast cancer) would recur or relapse after the standard initial treatment and over 5% of all breast cancer cases are diagnosed initially with stage IV disease (*de novo* stage IV disease) (3, 4). Unfortunately, even though huge improvements were observed in survival with the help of new drugs and appropriate therapeutic strategies in recent years, ABC (advanced breast cancer) is still incurable (5–8).

HR+ (hormone receptor-positive)/HER2- (human epidermal growth factor 2 negative) breast cancer accounts for ~70% of the total diagnosed BC cases worldwide, in which biological characteristics and prognosis are distinctly different from other subtypes (HER2 positive subtype/triple-negative subtype) (9). This subtype of BC shows sensitivity to anti-hormone therapy but is resistant to anti-HER2 treatment or immunotherapy. The ESMO (European Society for Medical Oncology) and NCCN (National Comprehensive Cancer Network) guidelines have provided evidence-based targeted optimizing recommendations for HR+/HER2- BC and HR+/HER2- ABC and descriptions of their clinicopathological features (10, 11).

Several models have been constructed and used for EBC prognostic prediction and treatment options (12–14). In addition, some factors, including molecular phenotypes, metastatic sites, stages previously diagnosed, previous therapy, and MFIs (metastatic-free intervals) could influence the survival of ABC patients and may be helpful to form an individualized therapeutic solution. It is also worth noting that few predictive models have been established and validated to evaluate specifically potential predictive factors for HR+/HER2- subtype BC. Furthermore, to date, no study has been published concerning the multivariable survival analysis in HR+/HER2- subtype ABC, which is the most prevalent subtype in all cases and is reported to be associated with commonly better outcomes and different factors influencing survival (10, 15–18).

In this study, we collected data from 1,671 HR+/HER2- ABC from different medical centers in China to investigate survival risk factors. At the same time, we intended to establish a comprehensive and practical nomogram to predict patient outcomes using this epidemiology, clinicopathological, and survival results data.

## Methods

### Data Collection and Patient Selection

The study was a hospital-based multicenter retrospective study conducted from January 1, 2012 to December 31, 2014. A total of 3,577 ABC patients from 21 hospitals covering seven geographic regions were involved in this study. The Chinese Academy of Medical Science cancer hospital was the lead center for the overall coordination of this research. To minimize selection bias, all enrolled institutions were given a random number of months to make the study operable with a representative selection. A designed questionnaire was assigned to each patient to obtain demographic and clinical variables. Trained physicians extracted the information from these ABC patients within the assigned months. If the number of inpatients were fewer than intended number, more cases would be added during the subsequent months until the total for the year reached the target quantity. Due to the Spring Festival, the months of January and February were excluded from the randomization.

In our study, the inclusion criteria were as follows (1): diagnosed with advanced breast cancer from January 1, 2012 to December 31, 2014, including *de novo* stage IV disease and relapsed disease (2); complete medical information including age at diagnosis, ER (estrogen receptor) or PR(progesterone receptor) status, HER2 status, distant metastasis sites, local therapy, treatment strategy (3); stage of initial diagnosis and treatment and MFI (metastatic-free interval) were necessary for patients with relapsed disease; and (4) HR positive and HER2 negative. The exclusion criteria were as follows (1): no available medical record and coexisting cancers (2); positive HER2 status; (3) both ER and PR status negative; and (4) TNBC(triple-negative breast cancer).

### Clinical Data

The status of ER, PR, and HER2 were mainly dependent on the pathology results of surgery or puncture biopsy. Hormone receptor status, including ER and PR, were assessed by IHC (immunohistochemistry) and HER2 was determined by either IHC or FISH (fluorescent *in-situ* hybridization). ER/PR positive was defined as ≥1% of cancer cell staining and cancers with 1%-10% of ER expression were considered ER-low-positive. Either ER or PR positive was regarded as HR-positive. An HER2 IHC score of 0; 1+ or none gene amplified by FISH would be defined as HER2 negative. If multiple biopsy results were inconsistent and one of the results was HR positive, we still considered this case to be an HR-positive tumor. Unlike HR status, when HER2 was detected overexpressed by re-biopsy after metastasis, this case would be defined as HER2 positive ABC disease and excluded from our study. The clinical stage was classified based on the 8^th^ AJCC (American Joint Committee on Cancer) TNM staging system.

Efficacy evaluation was measured by RECIST 1.1(Response Evaluation Criteria in Solid Tumors). MFI was the time between initial diagnosis of BC and confirmed recurrence or metastasis. Therapy lines were counted from the first treatment option after the ABC diagnosis. Regardless of the reason for the termination of previous therapy, altered treatment regimen received was considered as the therapy in next-line.

### Statistical Analysis

The primary endpoint was OS which was defined as the months from cancer metastasis or recurrence to death or last follow-up. We deleted variables with missing values greater than 30 percent to minimize biases. Univariable Cox regression analysis was performed to evaluate the significance of the association between clinical parameters and patient survival using the “survival” R package. Variables with a *p*-value less than 0.05(in the univariable case) from the univariable Cox-regression analysis were incorporated into a multivariable Cox regression model to identify independent prognostic factors with the help of the “survival” R package. From the univariable and multivariable Cox regression results, we developed a nomogram to effectively predict the 2-year, 3-year, 5-year survival probability of the patients using the “rms” R package. The discriminative ability of nomograms was evaluated by the AUC (the areas under the curve) of the ROC (receiver operating characteristic) curve through the “riskRegression” packages in the R software. Additionally, we employed a 5-fold and 200 time cross-validation approach to assess the performance of the nomogram on the validation cohort using the “caret” and “riskRegression” R packages. Calibration plots visually measured the closeness of the actual condition to the nomogram-predicted condition, the calibration plots of the training, and validation cohorts were produced using the “rms” R package.

We constructed the nomogram of the OS prediction model based on the multivariable Cox regression to categorize the patients into high-risk, medium-risk and low-risk groups using the “stats” (predict function) and “survival” R package. In the nomogram of the OS prediction model, the risk factor scores were added together to obtain a total score and the value corresponding to the total score was used to predict a patient’s 2-, 3-, and 5-year OS. Kaplan-Meier survival curve analysis with log-rank test and curve between three groups was conducted and plotted using the “survival” and “survminer” R packages. All data analysis was conducted using R software (4.1.2), and the parameters and functions associated with the R package are illustrated in **Table S1**.

## Results

### Characteristics of the Study Population

In our study, 1,671 patients identified with HR+/HER2- ABC disease were extracted from the original 3,577 ABC patients chosen between January 1 2012 and December 31 2014 included in our study. The detailed baseline clinicopathological features are documented in **Table 1**. We randomly split the dataset into a training cohort (1,155) and a validation cohort (495). Participants’ median age was 45.0 (range 20-89) years old; 33.1% (547) of patients were over 50 years old; 72% (1,190) of patients were dead at the end of follow-up time; the median OS and MFI were 26 months and 27 months, respectively; and 2-, 3- and 5-year OS rates were 54%, 34%, and 11%, respectively. As for ER/PR status, 77.7% (1,234) of patients were ER positive and 58% of patients were PR positive. Regarding the recurrence pattern, only 9% (148) patients experienced local-regional recurrence alone and 91% (1,489) patients had distant metastases, including 18.7% (302) had distant lymph node metastasis, 36.7% (595) had bone metastasis, 7.2% (117) had liver metastasis, 3.4% (55) had brain metastasis, 2.7% (44) had soft tissue metastasis, and 29.8% (482) had lung metastasis. During the therapy scheme, 53% (838) and 47% (743) of patients underwent first-line endocrine therapy and chemotherapy, respectively; 48.2% (341) and 51.8% (367) received second-line endocrine therapy and chemotherapy, respective; and 41% (642) patients accepted local therapy.

**Table 1:** Clinicopathological features of patients in the training and validation cohorts.

	Train Cohort	N		Validation Cohort	N
	N = 1155			N = 495	
Age		1155	Age		495
≤50	776 (67.2%)		≤50	327 (66.1%)	
>50	379 (32.8%)		>50	168 (33.9%)	
Grade		529	Grade		238
1	20 (3.78%)		1	7 (2.94%)	
2	322 (60.9%)		2	140 (58.8%)	
3	187 (35.3%)		3	91 (38.2%)	
ER status		1111	ER status		478
Positive	863 (77.7%)		Positive	371 (77.6%)	
Negative	248 (22.3%)		Negative	107 (22.4%)	
PR status		995	PR status		413
Positive	571 (57.4%)		Positive	246 (59.6%)	
Negative	424 (42.6%)		Negative	167 (40.4%)	
Re-biopsy after relapse		1065	Re-biopsy After relapse		453
No	521 (48.9%)		No	221 (48.8%)	
Yes	544 (51.1%)		Yes	232 (51.2%)	
Pathologic T stage		710	Pathologic T stage		310
T1	189 (26.6%)		T1	86 (27.7%)	
T2	418 (58.9%)		T2	180 (58.1%)	
T3	68 (9.6%)		T3	38 (12.3%)	
T4	35 (4.9%)		T4	6 (1.9%)	
Pathologic N stage		1155	Pathologic N stage		495
N0	500 (43.3%)		N0	224 (45.3%)	
N1	254 (22.0%)		N1	112 (22.6%)	
N2	224 (19.4%)		N2	91 (18.4%)	
N3	177 (15.3%)		N3	68 (13.7%)	
MFI (m)	Median 27 (0–360) months	1155	MFI (m)	Median 27 (0-216) months	495
Recurrence Pattern		1145	Recurrence Pattern		492
Locoregional Recurrence only	109 (9.5%)		Locoregional Recurrence only	39 (7.9%)	
Distant metastasis	1036 (90.5%)		Distant metastasis	453 (92.1%)	
First-line therapy option		1108	First-line therapy option		473
Endocrine therapy	589 (53.2%)		Endocrine therapy	249 (52.6%)	
Chemotherapy	519 (46.8%)		Chemotherapy	224 (47.4%)	
Second-line therapy option		496	Second-line therapy option		212
Endocrine therapy	229 (46.2%)		Endocrine therapy	112 (52.8%)	
Chemotherapy	267 (53.8%)		Chemotherapy	100 (47.2%)	
Local therapy		1103	Local therapy		469
No	653 (59.2%)		No	277 (59.1%)	
Yes	450 (40.8%)		Yes	192 (40.9%)	
Participate in clinical studies		868	Participate in clinical studies		380
No	666 (76.7%)		No	292 (76.8%)	
Yes	202 (23.3%)		Yes	88 (23.2%)	
Local recurrence		1137	Local recurrence		489
No	662 (58.2%)		No	293 (59.9%)	
Yes	475 (41.8%)		Yes	196 (40.1%)	
Distant Lymph node metastasis		1135	Distant Lymph node metastasis		486
No	924 (81.4%)		No	395 (81.3%)	
Yes	211 (18.6%)		Yes	91 (18.7%)	
Bone metastasis		1134	Bone metastasis		486
No	722 (63.7%)		No	303 (62.3%)	
Yes	412 (36.3%)		Yes	183 (37.7%)	
Liver metastasis		1134	Liver metastasis		486
No	904 (79.7%)		No	389 (80.0%)	
Yes	230 (20.3%)		Yes	97 (20.0%)	
Brain metastasis		1134	Brain metastasis		486
No	1096 (96.6%)		No	469 (96.5%)	
Yes	38 (3.4%)		Yes	17 (3.5%)	
Soft tissue metastasis		1134	Soft tissue metastasis		486
No	1101 (97.1%)		No	475 (97.7%)	
Yes	33 (2.91%)		Yes	11 (2.26%)	
Lung metastasis		1134	Lung metastasis		486
No	808 (71.3%)		No	330 (67.9%)	
Yes	326 (28.7%)		Yes	156 (32.1%)	
Other sites metastasis		1134	Other sites metastasis		486
No	1014 (89.4%)		No	436 (89.7%)	
Yes	120 (10.6%)		Yes	50 (10.3%)	
Number of metastatic sites	Median 1 (0-6)	1155	Number of metastatic sites	Median 1 (0-5)	495
Best efficacy of First-line therapy		1086	Best efficacy of First-line therapy		458
CBR	1001 (92.2%)		CBR	424 (92.6%)	
No CBR	85 (7.8%)		No CBR	34 (7.4%)	
OS		965	OS		406
≥2years	512(53%)		≥2years	225(55%)	
≥3years	324(34%)		≥3years	136(33%)	
≥5years	132(14%)		≥5years	50(12%)	

CBR (Clinical benefit rate) CR+PR*+SD≥6 months; OS, overall survival; N, Numbers; PR, Progesterone Receptor; ER, Estrogen Receptor; CR, Complete response; PR*, Partial response; SD, Stable disease; MFI, metastatic-free interval; m, months; N, Numbers.

### Prognostic Factors Selection of Overall Survival

According to a univariable analysis result of the training cohort, twelve variables are associated with OS in HR+/HER2- ABC patients. The variables are as follows: ER status, pathological T stage after the initial operation, MFI, recurrence pattern, 1st line therapy option, 2nd line therapy option, whether local therapy to metastatic sites was chosen, distant lymph nodes metastasis, liver metastasis, lung metastasis, number of metastatic sites, and whether re-biopsy was performed after recurrence or relapse (**Table 2**). We performed a multivariable analysis using all the above variables and identified ER status, MFI, first-line therapy option, the number of metastatic sites, and whether local therapy to metastatic sites as the five factors that, independently, could predict OS in HR+/HER2- ABC patients (**Table 2**).

**Table 2:** Univariable and Multivariable cox regression analyses of overall survival in the training cohort.

Univariable			Multivariable		
Characteristic	HR (95% CI)	*P* value	Characteristic	HR (95% CI)	*P* value
Age			Age		
≥50	Reference		≥50		
≤50	0.99 (0.85- 1.14)	0.85	≤50		
Grade			Grade		
1	Reference		1		
2	1.07 (0.55-2.09)	0.84	2		
3	1.07 (0.54-2.10)	0.85	3		
ER status			ER status		
Positive	Reference		Positive	Reference	
Negative	1.29 (1.08-1.53)	0.004	Negative	1.24 (1.02-1.50)	0.030
PR status					
Positive	Reference				
Negative	1.10 (0.94-1.28)	0.25			
Pathologic T stage					
T1	Reference				
T2/T3/T4	1.39 (1.12-1.73)	0.003			
Pathologic N stage					
N0	Reference				
N1	0.99 (0.82-1.19)	0.90			
N2	1.08 (0.89-1.31)	0.46			
N3	1.20 (0.98-1.49)	0.083			
MFI(m)	0.99 (0.99-1.00)	<0.001	MFI(m)	0.99 (0.99-1.00)	<0.001
Recurrence Pattern			Recurrence Pattern		
Locoregional Recurrence only	Reference		Locoregional Recurrence only	Reference	
Distant metastasis	1.69 (1.33-2.16)	<0.001	Distant metastasis	1.32 (0.95-1.84)	0.093
First-line therapy option			First-line therapy option		
Endocrine therapy	Reference		Endocrine therapy	Reference	
Chemotherapy	1.21 (1.04-1.40)	0.011	Chemotherapy	1.30 (1.10-1.55)	0.002
Second-line therapy option					
Endocrine therapy	Reference				
Chemotherapy	1.59 (1.28-1.97)	<0.001			
Local therapy			Local therapy		
No	Reference		No	Reference	
Yes	0.58 (0.50-0.68)	<0.001	Yes	0.62 (0.52-0.75)	<0.001
Participate in clinical studies			Participate in clinical studies		
No	Reference		No		
Yes	0.93 (0.77-1.13)	0.46	Yes		
localrecurrence					
No	Reference				
Yes	0.87 (0.75-1.01)	0.064			
Distant Lymph node metastasis			Distant Lymph node metastasis		
No	Reference		No	Reference	
Yes	1.31 (1.09-1.57)	0.004	Yes	1.02 (0.80-1.30)	0.9
Bone metastasis					
No	Reference				
Yes	1.14 (0.99-1.33)	0.078			
Liver metastasis			Liver metastasis		
No	Reference		No	Reference	
Yes	1.43 (1.20-1.70)	<0.001	Yes	1.06 (0.80-1.40)	0.7
Brain					
No	Reference				
Yes	1.08 (0.72-1.61)	0.71			
Soft tissue metastasis					
No	Reference				
Yes	0.91 (0.60-1.40)	0.68			
Lung metastasis			Lung metastasis		
No	Reference		No	Reference	
Yes	1.22 (1.04-1.42)	0.014	Yes	0.92 (0.70-1.20)	0.5
Other sites metastasis					
No	Reference				
Yes	1.31 (1.04-1.64)	0.021			
Number of metastatic sites	1.21 (1.13- 1.30)	<0.001	Number of metastatic sites	1.171 (0.005-1.30)	0.005
Best efficacy of First-line therapy					
CBR	Reference				
No CBR	0.97 (0.83-1.12)	0.68			
Re-biopsy after relapse			Re-biopsy after relapse		
No	Reference		No	Reference	
Yes	0.84 (0.73-0.98)	0.027	Yes	1.09 (0.92-1.30)	0.3

CBR (Clinical benefit rate) CR+PR*+SD≥6 months; OS, Overall survival; HR, Hazard ratio; CI, Confidence interval; PR, Progesterone Receptor; ER, Estrogen Receptor; CR, Complete response; PR*, Partial response; SD, Stable disease; MFI, metastatic-free interval; m, months.

### Nomogram Construction and Validation of Overall Survival

**Figure 1:**
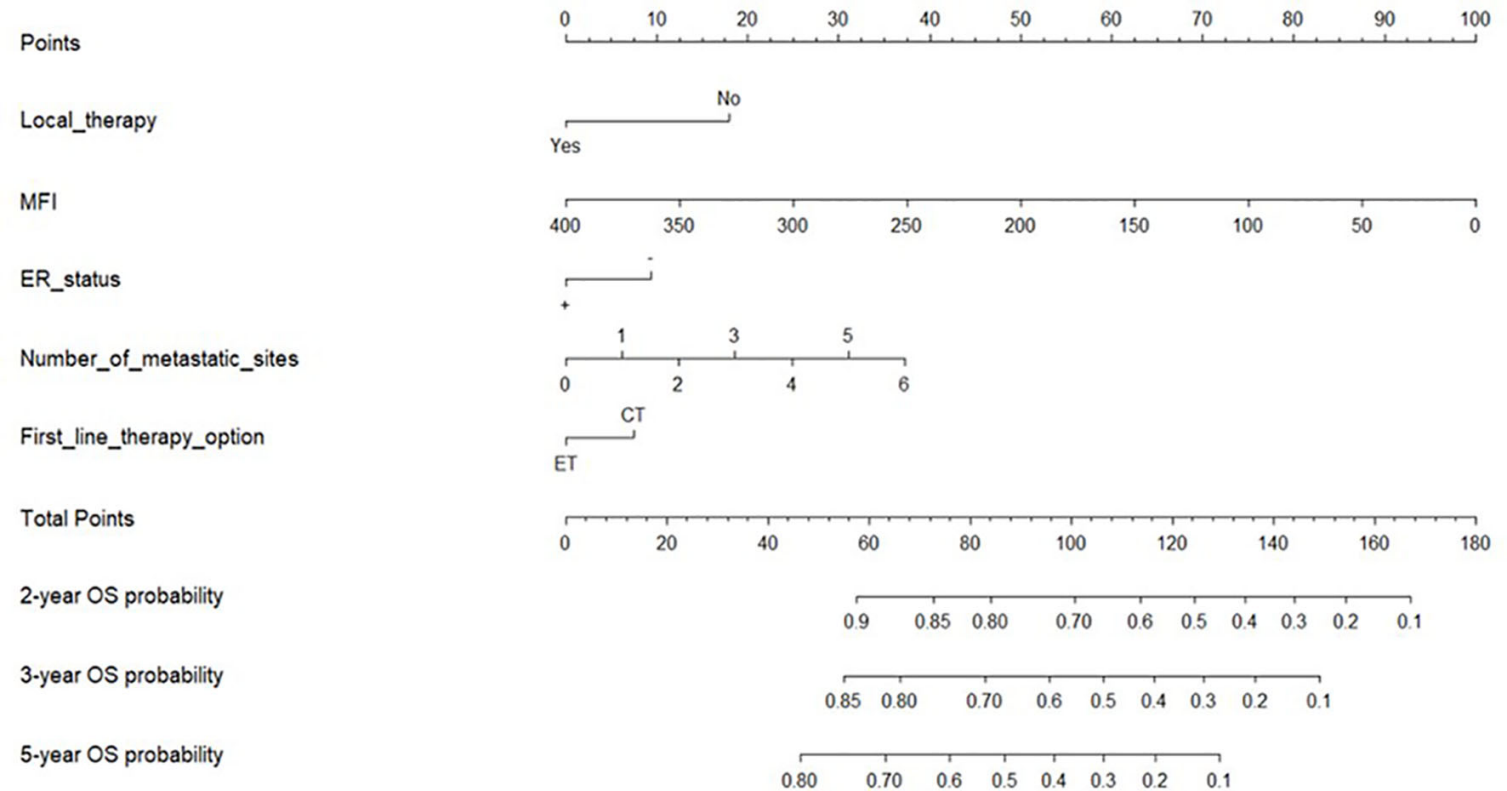
Nomogram for predicting the 2-year, 3-year, and 5-year overall survival. Notes: Nomogram used when summing the points identified at top scale for each of the five independent variables. This summed point score identified on total point scale was used to determinate 2-, 3- and 5-year overall survival (OS) probability of advanced breast cancer patients (ABC). MFI, metastatic-free interval; OS, overall survival; ER status, positive (+), negative (-); ET, Endocrine Therapy; CT, Chemotherapy.

**Figure 2:**
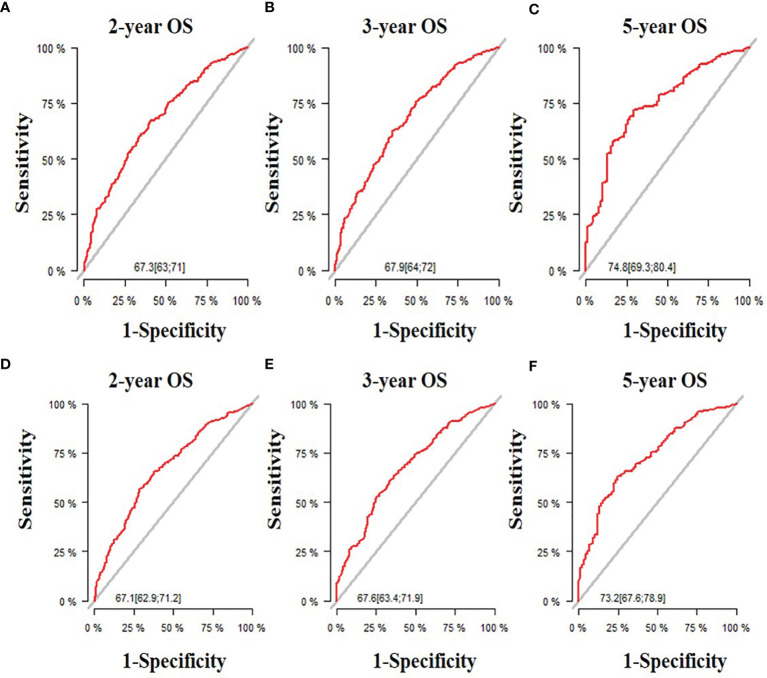
The receiver operating characteristic (ROC) curve and area under the ROC curve. **(A)** Predicting 2-year OS in the training cohort; **(B)** Predicting 3-year OS in the training cohort; **(C)** Predicting 5-year OS in the training cohort; **(D)** Predicting 2-year OS in the validation cohort;**(E)** Predicting 3-year OS in the validation cohort; **(F)** Predicting 5-year OS in the validation cohort.

**Figure 3:**
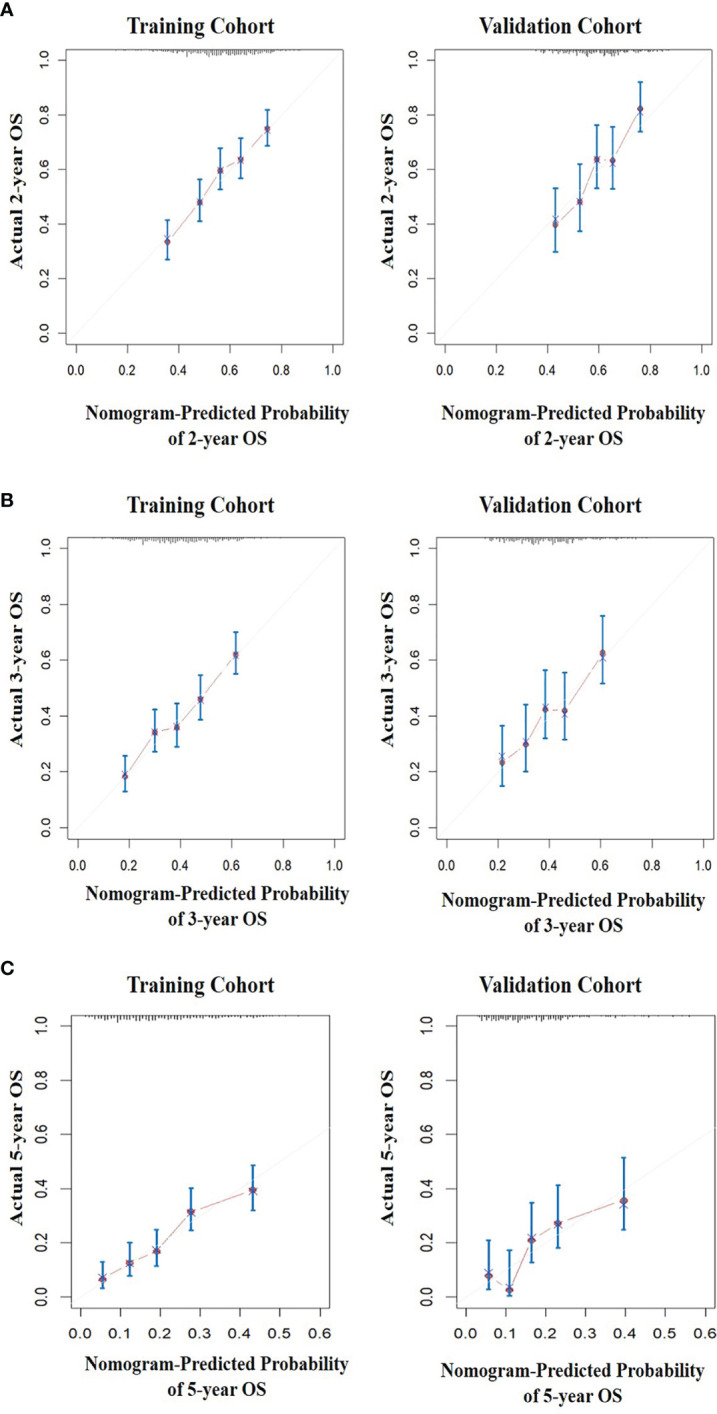
The calibration curves for predicting the survival of patients. **(A)** The 2-year OS prediction in the training and validation cohort after diagnosis. **(B)** The 3-year OS prediction in the training and validation cohort after diagnosis. **(C)** The 5-year overall survival prediction in the training and validation cohort after diagnosis.

All five significant prognostic factors identified in the multivariable Cox model in the training cohort were used to develop the predictive nomogram for estimating 2-, 3- and 5-year OS probability of HR+/HER2- ABC patients. Each variable was given a weighted value score on the scale axis to imply its contribution to the survival prognosis (**Figure 1**). Furthermore, we used the AUC of the ROC curve to evaluate the predictive accuracy of the nomogram. As illustrated in **Figure 2**, in the training cohort, the AUC of each 2-, 3- and 5-year of the nomogram model was 0.673, 0.679, 0.748, respectively (**Figures 2A–C**), suggesting a satisfying predicted outcome. Moreover, the nomogram model was validated by an internal test cohort with 495 participants. The validation cohort results also exhibited good discrimination with an AUC of 0.671 in predicting a 2 year OS probability, an AUC of 0.676 in predicting a 3 year OS probability, and an AUC of 0.732 in predicting a 5 year OS probability (**Figures 2D–F**). Furthermore, we employed a 5-fold cross-validation and 200 time approach to assess the performance of the nomogram on the validation cohort. The result showed that the mean AUC of each 2-, 3- and 5-year of the nomogram model for the validation cohort was 0.657, 0.645, 0.706, respectively (**Figure S1**). Conversely, to further estimate the nomogram’s accuracy, the plotted calibration showed good coordination between predicted and actual survival in 2-, 3- and 5-year outcomes, among the training and validation cohort (**Figure 3**).

### Risk Stratifications With the New Nomogram

**Figure 4:**
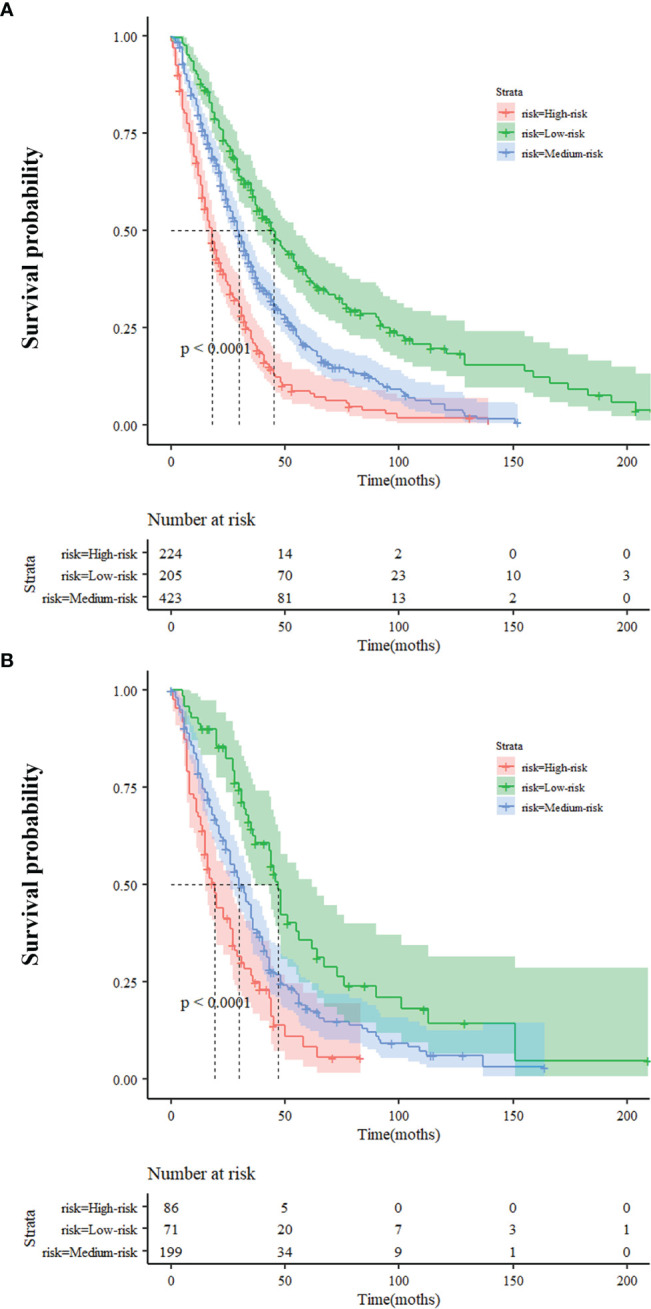
Survival probability of nomogram-based stratification of overall population **(A)** Training Cohort **(B)** Validation Cohort.

We divided patients from the training cohort into three subgroups based on the predicted risk score of the nomogram model for OS: low-risk (≤0.7), medium-risk (0.7-1.3), and high-risk (≥1.3). Survival analysis for OS showed a significant difference between three subgroups (*p<*0.001) (**Figure 4A**), and the median OS of the low-, medium-, and high-risk groups were 45, 30, and 18 months, respectively. The low-risk, medium-risk, and high-risk had a 70%, 54.4%, and 35% 2-year OS; a 51.7%, 32.9%, and 17.4% 3-year OS; and a 26.8%, 12.1%, and 4.9% 5-year OS, respectively. In the validation cohort, notable differences were also observed among three subgroups with a median OS of 47, 30, and 19 months for low-risk, medium-risk, and high-risk groups, respectively (*p<*0.001) (**Figure 4B**). Similarly, the low-risk, mediumrisk, and high-risk groups had a 77.5%, 55.8%, and 35% 2-year OS; a 49.3%, 35.7%, and 18.6% 3-year OS; and a 22.5%, 19.8%, and 3.5% 5-year OS, respectively.

## Discussion

The cause, occurrence, and development of malignant tumors are complicated and multifactorial and every factor plays only a limited role throughout the whole course of the disease. A nomogram is a comprehensive statistical model based on multivariable analysis that combines all statistically significant variables to improve accuracy and make the outcomes more intuitive. It has been reported that, in diverse cancers, a nomogram shows its excellent predictive value in evaluating oncology risk, chosen therapeutics and medicine, and survival outcome prediction (19–21). Our study collected patient information from 1,671 HR+/HER2- ABC patients from all subtypes from the overall 3,577 ABC patients. After the screening and statistical analysis, we identified ER status, MFI, first-line therapy options, locoregional therapy, and the number of metastatic sites as independent prognostic factors for OS of HR+/HER2- ABC patients. Interestingly, these prognostic factors extracted from our analysis showed partial consistency with current consensus and guidelines, whereas, in some aspects, differing from other predictive models for ABC patients’ survival (10, 15, 18, 22). R. Largillier et al. (16) used a Cox proportional hazards model to identify age, the number of positive lymph nodes, adjuvant chemotherapy, and site of metastasis as independent predictors of stage IV BC patients’ OS. Furthermore, liver metastasis, brain metastasis, lung metastasis, high histological grade, and large tumor size were reported to be associated with poor survival (15, 18, 23). However, all factors mentioned above did not have a prognostic effect on HR+/HER2- ABC patients’ OS in our research results. Differences in the results between our study and previous studies reflected that ABC is a kind of heterogeneous disease and the molecular subtype of ABC could be a significant independent predictor (24). We excluded HER2 positive BC and TNBC to reveal more specific and targeted prognostic factors for ABC of HR+/HER2- subtype.

Generally, it has been agreed that ER is a powerful bio-marker of sensitivity to endocrine therapy and is a vital prognosis sign of good survival (25–27). Nevertheless, whether the positivity of PR status alone has an equivalent effect in predicting a response to endocrine treatment remains controversial. Several studies suggest that both PR and ER expression were biomarkers of sensitivity to endocrine therapy, while recently, some arguments have emerged for the morphological and molecular features of ER-/PR+ BC are close to triple-negative BC (27, 28). A long-term consensus suggests that PR localized downstream of ER-mediated signaling pathways and its status to some extent driven by ER status. To date, there is little cohort research or robust evidence to characterize ER-/PR+ tumors. Our study found that ER and PR status were both statistically significant in univariable analysis, but only ER positive showed its power to predict better survival in multivariable analysis.

Unlike prior predictive models, the months of MFI and the number of metastatic sites were included in our nomogram model as continuous instead of categorical variables. We could observe, in this case, a worsening of survival accompanied by increased metastatic tumor burden and earlier recurrence. HR+ breast cancer is characterized by favorable survival, but a higher risk of long-term recurrence than HER2 over-expression and TNBC subtypes. In the data we collected, the time between initial diagnosis of malignancies and recurrence or relapse could be up to 360 months. Clinically relevant and validated prognostic models for MBC (metastatic breast cancer) highlighted the importance of MFI and could reflect the multiparametric variability of the disease. A shorter MFI is frequently accompanied by more aggressiveness and higher mortality, especially in patients who underwent adjuvant therapy. Similar to the findings reported, MFI showed its directed prognosis in relapse HR+/HER2- ABC in our analysis, with no difference with other subtypes of ABC (3, 16, 29).

Evidence-based guidelines have provided some recommendations for optimizing the management of ABC. The first step is re-testing the receptor status of recurrent diagnostic tissue. The discordance of HR and HER2 levels between primary and recurrent diseases have been reported at rates ranging from 3.4%–60% (10, 11, 30, 31). For HR+ breast cancer patients, the choice of endocrine therapy is reasonable as long as there has been a positive test result, regardless of the consistency of multiple biopsies. In contrast, anti-HER2 targeted treatment should be initiated immediately no matter when the status of HER2 has been tested overexpressed after re-biopsy. Once the HER2 status turns positive, the patient is considered a HER2+ patient and must be removed from the study population. That is one of the bases we set as our inclusive and exclusive criteria of HR+/HER2- ABC for selecting the population in our study. It is unexpected that re-biopsy did not have statistical significance in multivariable analysis (**Table 2**). This discrepancy between our result and reported outcomes may be caused by the difference in guideline recommendations and real-world clinical practices. Existing guidelines recommend ET (endocrine therapy) as the preferred first-line therapeutic option for HR+ ABC patients without visceral crisis or rapid progression diseases (10, 11). In our study, the choice of ET as first-line treatment was also found to indicate a prolonged OS of HR+/HER2- ABC. However, in real-world medical practices reflected in the collected data, about half of HR+/HER2- ABC patients accepted CT (chemotherapy) as the first-line and second-line systemic treatment, respectively. The reasons affecting treatment options are complex. In any case, although the multivariable analysis showed no statistical significance, the results of re-biopsy in the univariable analysis in this study are still valuable for the recommended role of diagnostic tools in clinical practices.

In addition, there are a number of locoregional treatment options available for ABC disease, such as: surgery, radiotherapy, radiofrequency ablation, and interventional therapy. These appropriate local therapeutic strategies are no longer considered a way to bring the patients to a “tumor-free state”, but rather as a supplement to systemic treatment. To date, no consensus has been reached on when and how to choose locoregional treatment and a multi-disciplinary team is extraordinarily needed at this point. Proper local treatment could alleviate patients’ pain, quickly control potentially life-threatening complications, remove drug-resistant lesions, and give patients opportunities to receive more lines of systemic therapy.

Nevertheless, several limitations in this current study are worth noting. Firstly, patients in both training and validation cohorts were retrospectively chosen from comprehensive multicenter databases. Missing clinical data and a loss of follow-up information would be inevitable during the collection process, leading to the bias of the multivariable analysis. Secondly, all research data collection ended on December 31, 2014, at which time, CDK inhibitors, which are considered as somewhat of a breakthrough drug, were not yet approved to be used in HR+/HER2- ABC. This class of drugs has re-written the survival rate of patients with of HR+ BC disease and has continued to develop in recent years. All the above deficiencies might affect the predictive capacities of this nomogram. Our predictive model has provided several influential survival factors and some guidance for further prospective HR+/HER2- subtype ABC studies. However, it may not be appropriate to guide current clinical practice until the results of larger prospective trials are released.

## Conclusion

In general, we made the first attempt to construct a novel prediction nomogram for estimating OS of HR+/HER2- subtype ABC disease. Our analysis selected ER status, MFI, first-line therapy option, locoregional therapy, and the number of metastatic sites from a wide range of epidemiology and clinicopathological features to predict HR+/HER2- ABC survival. The nomogram provided a more straightforward method of insight into this subtype ABC patients’ future outcomes and, to a certain extent, assisted physicians in making the personalized therapeutic option.

## Data Availability Statement

The raw data supporting the conclusions of this article will be made available by the authors, without undue reservation.

## Ethics Statement

The studies involving human participants were reviewed and approved by the relevant institutional review board, the Ethics Committee/National Cancer Center/National Clinical Research Center for Cancer/Cancer Hospital, Chinese Academy of Medical Sciences, and Peking Union Medical College. The patients/participants provided their written informed consent to participate in this study.

## Author Contributions

Data collection and analysis, Data interpretation, Manuscript writing, Final approval of the manuscript: YZ. Development of methodology, Data collection and analysis, Data interpretation, Manuscript writing, Final approval of the manuscript: JW. Conception and design, Data collection and analysis, Data interpretation, Manuscript writing, Final approval of the manuscript: BX. All authors are in agreement with the content of the manuscript.

## Conflict of Interest

The authors declare that the research was conducted in the absence of any commercial or financial relationships that could be construed as a potential conflict of interest.

## Publisher’s Note

All claims expressed in this article are solely those of the authors and do not necessarily represent those of their affiliated organizations, or those of the publisher, the editors and the reviewers. Any product that may be evaluated in this article, or claim that may be made by its manufacturer, is not guaranteed or endorsed by the publisher.

## Supplementary Material

Supplementary Figure 1 The average AUC of the cross-validation for the validation cohort.
