# Supplementary figures and images for: A Novel Prognostic Nomogram for Predicting Survival of Hormone Receptor-Positive and HER2 Negative Advanced Breast Cancer Among the Han-Population
Source: Front Oncol. 2022 Jul 1;12:918759. doi: 10.3389/fonc.2022.918759 (PMC9285102; doi:10.3389/fonc.2022.918759)

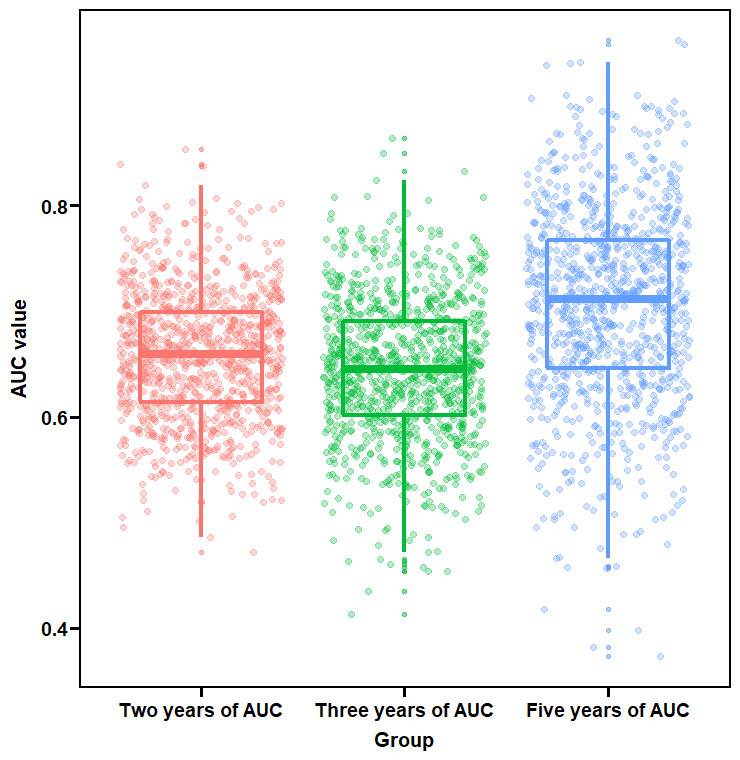

Supplement: Supplementary Figure 1 — The average AUC of the cross-validation for the validation cohort. [file Image_1.tif]
