# Supplementary material for: A Novel Prognostic Nomogram for Predicting Survival of Hormone Receptor-Positive and HER2 Negative Advanced Breast Cancer Among the Han-Population
Source: Front Oncol. 2022 Jul 1;12:918759. doi: 10.3389/fonc.2022.918759 (PMC9285102; doi:10.3389/fonc.2022.918759)
Supplement: Supplementary file 2 [file Table_1.docx]

**Table S1** The associated parameters of R package

| **Package** | **Function** | **Usage** | **Parameters** |
| --- | --- | --- | --- |
| Survival | Coxph | Univariate Cox regression analysis | Default |
|  |  | Multivariate Cox regression analysis |  |
|  | survfit | Create survival curves | Default |
| survminer | ggsurvplot | Drawing Survival Curves | Default |
| Rms | nomogram | Building nomogram model | Default |
|  | calibrate | Create calibrate plot | Cmethod=“KM”  B=1000  M=150/70 for Train cohort/Validation cohort |
| Caret | createMultiFolds | creates cross-validation split for the validate Cohort | K=5  Times=200 |
| Base | plot | Plot nomogram | Default |
| riskregression | Score | Score the predictive performance of the nomogram model | Plots=“Roc”  Metrics=“auc” |
|  | plotROC | Plot ROC Curve | Default |
| Stats | Predict | Predict event probabilities and risk stratifications for the nomogram | Type=“risk” |
